# Supplementary material for: Visuo-thermal congruency modulates the sense of body ownership
Source: Commun Biol. 2022 Jul 22;5:731. doi: 10.1038/s42003-022-03673-6 (PMC9307774; doi:10.1038/s42003-022-03673-6)
Supplement: Supplementary file 2 — Supplementary Information [file 42003_2022_3673_MOESM2_ESM.docx]

**Supplementary materials**

**Visuo-thermal congruency modulates the sense of body ownership**

Laura Crucianelli^1^ & H. Henrik Ehrsson^1^

^1^Department of Neuroscience, Karolinska Institutet, Stockholm, Sweden

**Experiment 1**

**Skin temperature data**

We compared the skin temperature of the left arm, left hand, and right hand before and after the tactile stimulation period in all the conditions and for all the outcome measures. For the embodiment analysis, a 2 (pre vs. post) x 3 (location) x 2 (congruency) x 2 (temperature) repeated-measures ANOVA revealed a main effect of location (F (2, 56) = 25.85, p < 0.01). Bonferroni corrected post hoc t tests (α = 0.017) showed that the temperature of the left arm (mean = 31.60; SD = 0.01) was significantly different from the temperature of the left hand (M = 29.45; SD = 0.05; *t*(33) = 8.43, *p* < 0.01) and right hand (M = 29.33; SD = 0.39; *t*(29) = 6.36, *p* < 0.01). No significant differences between the temperature of the left hand and of the right hand were found (*t*(28) = 0.35, *p* = 0.73). No other significant main effects or interactions were found.

For proprioceptive drift, a 2 (pre vs. post) x 3 (location) x 2 (congruency) x 2 (temperature) repeated-measures ANOVA revealed a main effect of location (F (2, 60) = 10.38, p < 0.01). Bonferroni corrected post hoc t tests (α = 0.017) showed that the temperature of the left arm (mean = 31.61; SD = 0.02) was significantly different from the temperature of the left hand (M = 29.96; SD = 0.11; *t*(37) = 4.92, *p* < 0.01) and right hand (M = 30.16; SD = 0.15;; *t*(30) = 3.51, *p* < 0.01). No significant differences between the temperature of the left hand and of the right hand were found (*t*(30) = 0.31, *p* = 0.76). No other significant main effects or interactions were found (see Supplementary Table 1).

There was a main effect of time (*F*(1, 30) = 4.40, *p* = 0.04), with the temperature post-stimulation (M = 30.60, SD = 0.75) being significantly higher than the temperature pre-stimulation (M = 30.55, SD = 0.79), regardless of condition. There was also a congruency x time significant interaction (*F*(1, 30) = 5.28, *p* = 0.03). However, Bonferroni corrected post hoc analyses (α = 0.025) revealed non-significant differences between pre and post-temperature for the congruent conditions (*t*(30) = -1.38, *p* = 0.18) and for the incongruent conditions (*t*(30) = -1.58, *p* = 0.12). No other significant main effects or interactions were found (see Supplementary Table 1).

Finally, for the thermal matching task, a 2 (pre vs. post) x 3 (location) x 2 (congruency) x 2 (temperature) repeated-measures ANOVA revealed a main effect of location (*F* (2, 54) = 22.63, *p* < 0.01). Bonferroni corrected post hoc t tests (α = 0.017) showed that the temperature of the left arm (mean = 31.64; SD = 0.04) was significantly different from that of the left hand (M = 29.52; SD = 0.09; *t*(32) = 8.50, *p* < 0.01) and right hand (M = 29.57; SD = 0.07; *t*(27) = 5.52, *p* < 0.01). No significant differences between the temperature of the left hand and of the right hand were found (*t*(27) = 0.22, *p* = 0.83). No other significant main effects or interactions were found (see Supplementary Table 1).

**Supplementary Table 1.** Skin temperature for the left arm, left hand and right hand registered pre- and post-tactile stimulation. We report the mean and (standard deviation) for the proprioceptive drift (PD) trial, for the subjective embodiment (Emb) task and for the thermal matching task (TMT) separately.

| Experiment 1 | | Pre-tactile stimulation | | | Post-tactile stimulation | | |
| --- | --- | --- | --- | --- | --- | --- | --- |
| TASK | CONDITION | Left arm | Left hand | Right hand | Left arm | Left hand | Right hand |
| PD | Congruent warm | 31.597  (0.225) | 30.059  (1.857) | 30.027  (2.064) | 31.590  (0.230) | 30.130  (1.834) | 30.162  (2.148) |
|  | Incongruent warm | 31.602  (0.232) | 29.897  (1.226) | 29.959  (2.283) | 31.602  (0.227) | 29.912  (1.318) | 30.055  (2.269) |
|  | Congruent cold | 31.632  (0.270) | 29.984  (1.282) | 30.338  (2.223) | 31.632  (0.274) | 30.016  (1.317) | 30.376  2.170 |
|  | Incongruent cold | 31.595  (0.215) | 29.772  (1.247) | 30.107  (2.189) | 31.620  (0.256) | 29.894  (1.263) | 30.268  (2.278) |
| Emb | Congruent warm | 31.590  (0.182) | 29.382  (1.226) | 29.368  (1.600) | 31.615  (0.239) | 29.381  (1.274) | 29.485  (1.504) |
|  | Incongruent warm | 31.592  (0.222) | 29.452  (1.245) | 28.404  (5.197) | 31.590  (0.223) | 29.400  (1.232) | 29.328  (1.579) |
|  | Congruent cold | 31.605  (0.257) | 29.517  (1.228) | 29.500  (1.654) | 31.606  (0.257) | 29.466  (1.202) | 29.527  (1.669) |
|  | Incongruent cold | 31.617  (0.264) | 29.462  (1.1447) | 29.445  (1.649) | 31.620  (0.263) | 29.506  (1.172) | 29.590  (1.676) |
| TMT | Congruent warm | 31.622  (0.238) | 29.516  (1.337) | 29.465  (1.759) | 31.637  (0.242) | 29.543  (1.275) | 29.522  (1.665) |
|  | Incongruent warm | 31.595  (0.184) | 29.393  (1.111) | 29.489  (1.772) | 31.603  (0.191) | 29.359  (1.168) | 29.604  (1.748) |
|  | Congruent cold | 31.662  (0.321) | 29.517  (1.219) | 29.621  (1.733) | 31.657  (0.322) | 29.613  (1.245) | 29.610  (1.720) |
|  | Incongruent cold | 31.687  (0.346) | 29.533  (1.233) | 29.637  (1.738) | 31.692  (0.349) | 29.630  (1.387) | 29.650  (1.765) |

**Correlational analyses across illusion measures**

Correlational analyses between the illusion measures showed no significant correlations between the illusion shift and the proprioceptive shift in any conditions (warm: *r_s_* = 0.10, *p* = 0.55; cold: *r_s_* = - 0.14, *p* = 0.38). There were no significant correlations between the proprioceptive shift and the thermal shift in any conditions (warm increasing: *r_s_* = 0.10, *p* = 0.55; warm decreasing: *r_s_* = 0.19, *p* = 0.23; cold increasing: *r_s_* = -0.03, *p* = 0.85; cold decreasing: *r_s_* = - 0.02, *p* = 0.96). Similarly, there were no significant correlations between the illusion shift and the thermal shift in any of the conditions (warm increasing: *r_s_* = -0.02, *p* = 0.88; warm decreasing: *r_s_* = -0.04, *p* = 0.78; cold increasing: *r_s_* = -0.14, *p* = 0.40; cold decreasing: *r_s_* = - 0.06, *p* = 0.74).

Spearman correlational analyses revealed a significant correlation between the BPQ scores and illusion shift in the cold condition (*r_s_* = 0.33, *p* = 0.04) but not in the warm condition (*r_s_* = 0.15, *p* = 0.37). There were no significant correlations between BPQ scores and proprioceptive shift (warm: *r_s_* = -0.22, *p* = 0.19; cold: *r_s_* = 0.23, *p* = 0.16). There were non-significant correlations between the BPQ scores and the thermal shift (warm increasing: *r_s_* = 0.15, *p* = 0.35 and decreasing: *r_s_* = 0.31, *p* = 0.053; cold increasing: *r_s_* = 0.13, *p* = 0.41 and decreasing: *r_s_* = 0.16, *p* = 0.32).

**Experiment 2**

**Skin temperature data**

As in Experiment 1, we compared the skin temperature for the left arm, left hand, and right hand before and after the tactile stimulation period in all the conditions and for all the outcome measures. For the embodiment, a 2 (pre vs. post) x 3 (location) x 2 (congruency) x 2 (temperature) repeated-measures ANOVA revealed a main effect of location (*F*(2, 58) = 31.82, *p* < 0.01). Bonferroni corrected post hoc t tests (α = 0.017) showed that the temperature of the left hand (M = 28.82; SD =0.05) was significantly different from that of the left arm (M = 31.64; SD = 0.03; *t*(32) = 813.98, *p* < 0.01) and right hand (M = 30.62; SD = 0.09; *t*(29) = -4.40, *p* < 0.01). No significant differences between the temperature of the left arm and of the right hand were found (*t*(29) = 2.48, *p* = 0.20. (Note that in this experiment, the RHI was eliminated in all conditions, so there was no reason to expect possible temperature changes related to the illusion of the kind reported by Moseley et al., 2008.)

There was also a main effect of time (F(1,29) = 11.63, p < 0.01), with the temperature post-stimulation (M = 30.34, SD = 1.23) being significantly lower than the temperature pre-stimulation (M = 30.37, SD =1.21), regardless of condition. There was also a location x time significant interaction (*F*(2, 58) = 4.23, *p* = 0.02). Bonferroni corrected post hoc analyses (α = 0.025) revealed significant differences between pre- and post-temperature for the left hand (*t*(32) = 3.25, *p* <0.01) and for the right hand (*t*(29) = 2.77, *p* = 0.01) but not for the left arm (*t*(32) = -0.14, *p* = 0.89). No other significant main effects or interactions were found (see Supplementary Table 2).

For proprioceptive drift, a 2 (pre vs. post) x 3 (location) x 2 (congruency) x 2 (temperature) repeated-measures ANOVA revealed a main effect of location (*F* (2,56) = 28.55, p < 0.01). Bonferroni corrected post hoc t tests (α = 0.017) showed that overall, the temperature of the left arm (mean = 31.64; SD = 0.01) was significantly different from that of the left hand (M = 29.53; SD = 0.04; *t*(32) = 12.67, *p* < 0.01) and right hand (M = 30.78; SD = 0.21; *t*(28) = 2.94, *p* < 0.01). The temperature of the left hand was also different from that of the right hand (*t*(28) = -3.73, *p* <0.01). No other significant main effects or interactions were found (see Supplementary Table 2).

Finally, for the thermal matching task, a 2 (pre vs. post) x 3 (location) x 2 (congruency) x 2 (temperature) repeated-measures ANOVA revealed a main effect of location (*F*(2,58) = 27.68, p < 0.01). Bonferroni corrected post hoc t tests (α = 0.017) showed that the temperature of the left hand (M = 29.10, SD = 0.03) was significantly different from the temperature of the left arm (M = 31.66, SD = 0.02; *t*(32) = 14.06, *p* < 0.01) and right hand (M = 30.77, SD = 0.05; *t*(29) = -4.28, *p* < 0.01). No significant differences between the temperature of the left arm and of the right hand were found (*t*(29) = 1.94, *p* = 0.06). No other significant main effects or interactions were found (see Supplementary Table 3).

**Supplementary Table 2.** Skin temperature for the left arm, left hand and right hand registered pre- and post-tactile stimulation. We report the mean and (standard deviation) for the proprioceptive drift (PD) trial, for the subjective embodiment (Emb) task and for the thermal matching task (TMT) separately.

| Experiment 2 | | Pre-tactile stimulation | | | Post-tactile stimulation | | |
| --- | --- | --- | --- | --- | --- | --- | --- |
| TASK | CONDITION | Left arm | Left hand | Right hand | Left arm | Left hand | Right hand |
| PD | Congruent synchronous | 31.636  (0.281) | 29.554  (1.039) | 30.557  (1.756) | 31.651  (0.294) | 29.606  (1.019) | 30.550  (1.821) |
|  | Congruent asynchronous | 31.633  (0.278) | 29.518  (0.974) | 31.040  (1.985) | 31.651  (0.289) | 29.497  (0.960) | 30.759  (1.607) |
|  | Incongruent synchronous | 31.658  (0.312) | 29.476  (0.888) | 30.570  (1.674) | 31.627  (0.274) | 29.533  (0.925) | 30.771  (2.116) |
|  | Incongruent asynchronous | 31.642  (0.293) | 29.500  (0.900) | 30.950  (2.079) | 31.648  (0.285) | 29.539  (0.897) | 31.037  (2.115) |
| Emb | Congruent synchronous | 31.633  (0.304) | 28.821  (1.132) | 30.647  (2.308) | 31.642  (0.299) | 28.764  (1.094) | 30.630  (2.299) |
|  | Congruent asynchronous | 31.673  (0.336) | 28.858  (1.150) | 30.573  (2.310) | 31.679  (0.339) | 28.815  (1.190) | 30.477  (2.356) |
|  | Incongruent synchronous | 31.600  (0.221) | 28.794  (1.122) | 30.568  (2.323) | 31.615  (0.236) | 28.748  (1.198) | 30.597  (2.319) |
|  | Incongruent asynchronous | 31.651  (0.302) | 28.876  (1.193) | 30.783  (2.195) | 31.627  (0.270) | 28.858  (1.189) | 30.663  (2.210) |
| TMT | Congruent synchronous | 31.627  (0.276) | 29.148  (1.091) | 30.732  (2.330) | 31.651  (0.288) | 29.085  (1.058) | 30.777  (2.270) |
|  | Congruent asynchronous | 31.648  (0.293) | 29.106  (0.956) | 30.850  (2.304) | 31.642  (0.292) | 29.070  (0.992) | 30.840  (2.254) |
|  | Incongruent synchronous | 31.682  (0.323) | 29.151  (1.015) | 30.739  (2.351) | 31.691  (0.340) | 29.076  (1.039) | 30.723  (2.373) |
|  | Incongruent asynchronous | 31.667  (0.326) | 29.121  (1.073) | 30.764  (2.272) | 31.664  (0.324) | 29.076  (1.079) | 30.752  (2.298) |

**Illusion questionnaire: Thermo-affective items**

Wilcoxon signed ranks tests showed no significant main effect of synchronicity (*Z* = -0.73, *p* = 0.48, mean synchronous = 0.89, SD = 1.07; mean asynchronous = 1.01, SD = 1.22) or congruency (*Z* = -1.73, *p* = 0.09, mean congruent = 0.79, SD = 1.31; mean incongruent = 1.12, SD = 1.33) on the *tactile pleasantness* reported. In terms of explicit cold perception, we found a non-significant main effect of synchronicity (*Z* = -0.57, *p* = 0.58, mean synchronous = 0.14, SD = 0.90; mean asynchronous = 0.02, SD = 1.09). However, there was a significant main effect of congruency (*Z* = -5.02, *p* < 0.01, mean congruent = 1.95, SD = 1.13; mean incongruent = -1.80, SD = 1.18) on *cold perception*; that is, participants explicitly reported the temperature of the felt touch according to the temperature that they were perceiving (24 °C in the congruent condition and 32 °C in the incongruent condition) regardless of the object that was touching the rubber hand. Finally, we found a non-significant main effect of synchronicity (*Z* = -0.26, *p* = 0.81, mean synchronous = -0.55, SD = 0.83; mean asynchronous = -0.38, SD = 1.01) on *warm perception*. However, there was a significant main effect of congruency (*Z* = -4.93, *p* < 0.01, mean congruent = -2.33, SD = 0.97; mean incongruent = 1.41, SD = 1.20) on warm perception, in the sense that touch was reported as warmer after the incongruent condition (seen cold, perceived neutral) compared to that after the congruent conditions (seen cold, felt cold).

**Analysis combining data from Experiments 1 and 2**

In an exploratory analysis, we combined the data from Experiment 1 and Experiment 2 for those conditions where the thermal stimulation was identical across the two experiments (see Figure 1 in the main manuscript). In particular, we were interested in comparing the congruent cold condition (i.e., participant feeling the touch at 24 °C on their skin while looking at the rubber hand being touched by an ice cube) across Experiment 1 (classic rubber hand illusion) and Experiment 2 (hand placed at 90°).

In terms of the subcomponents of embodiment, the results of the Wilcoxon signed ranks test revealed a significant main effect of experiment on the referral of touch scores (Z = -3.79, p < 0.01), with participants in Experiment 1 showing a higher level of referral of touch than participants in Experiment 2 (M_Exp 1_ = 1.72, SD = 1.87; M_Exp 2_ = 0.03, SD = 2.24). Similarly, there was a significant main effect of experiment on the ownership scores (Z = -4.33, p < 0.01), with participants in Experiment 1 showing a higher level of ownership towards the rubber hand than participants showed in Experiment 2 (M_Exp 1_ = 0.8, SD = 1.81; M_Exp 2_ = -1.39, SD = 1.90). No significant effect of experiment was found for the location item of the questionnaire (*Z* = -1.07, *p* =0.28; M_Exp 1_ = 2.18, SD = 1.41; M_Exp 2_ = 1.82, SD = 1.70).

Finally, the results of the Wilcoxon signed ranks test revealed a significant main effect of experiment on performance on the thermal matching task in the decreasing trials (Z = -2.75, p < 0.01; M_Exp 1_ = 1.25, SD = 2.06; M_Exp 2_ = 2.36, SD = 1.54) but not in the increasing trials (Z = -0.97, p = 0.33; M_Exp 1_ = 1.10, SD = 1.35; M_Exp 2_ = 1.58, SD = 1.79).


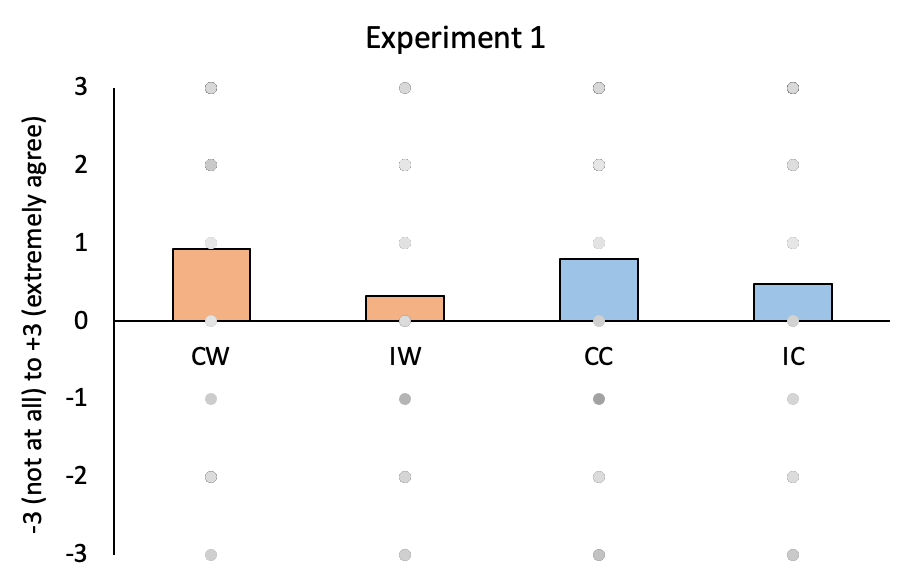

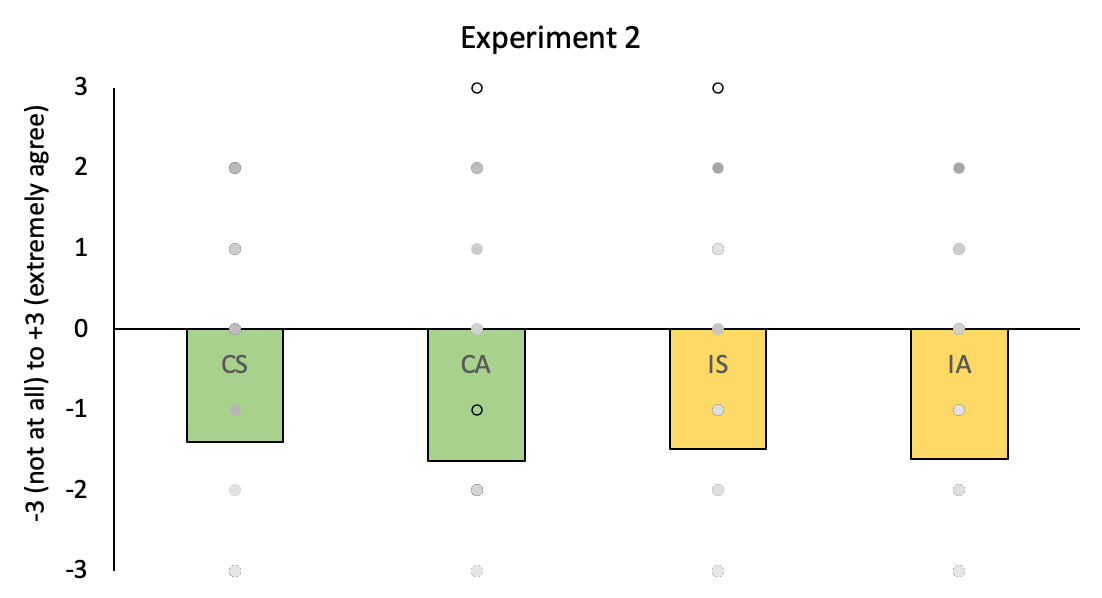


**Supplementary Figure 1.** Mean scores and individual data point distribution for the illusion composite score of the illusion questionnaire for Experiments 1 (CW = congruent warm; IW = incongruent warm; CC = congruent cold; IC = incongruent cold) and 2 (CS = congruent synchronous; CA = congruent asynchronous; IS = incongruent synchronous; IA = incongruent asynchronous). See also Figure 1a in the main text.


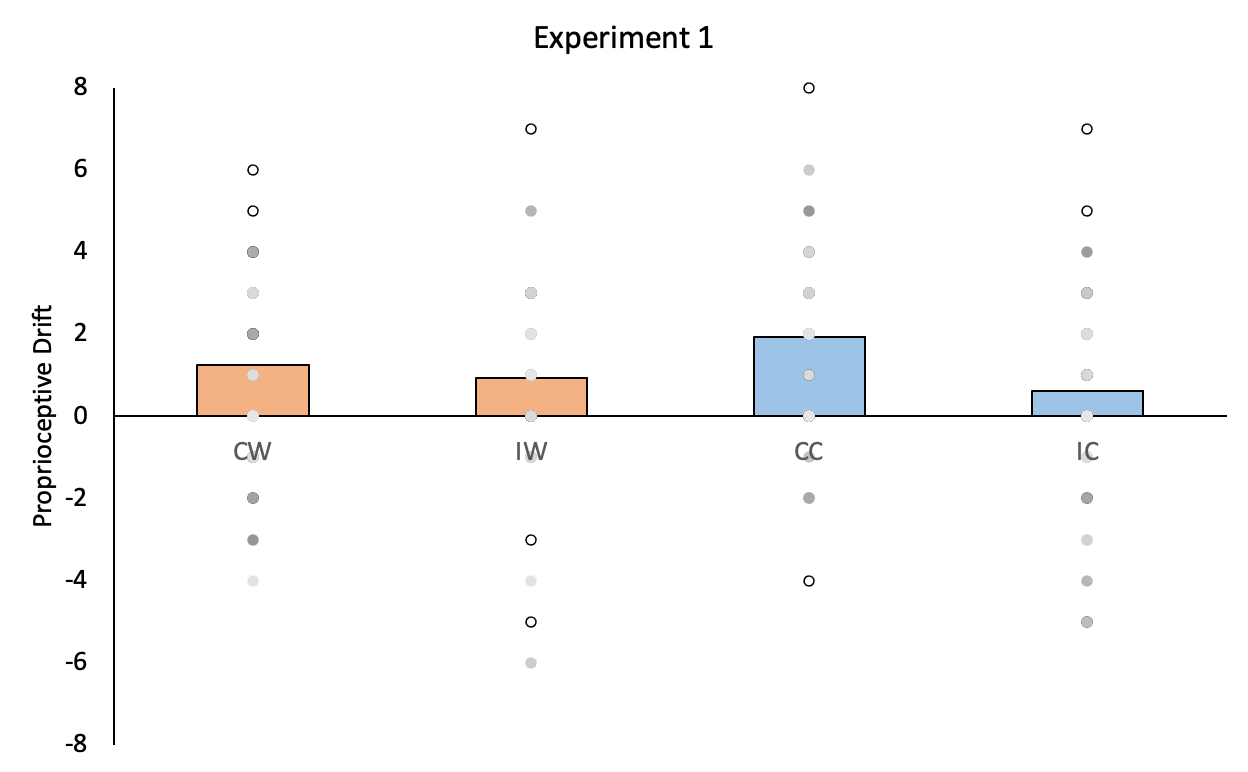

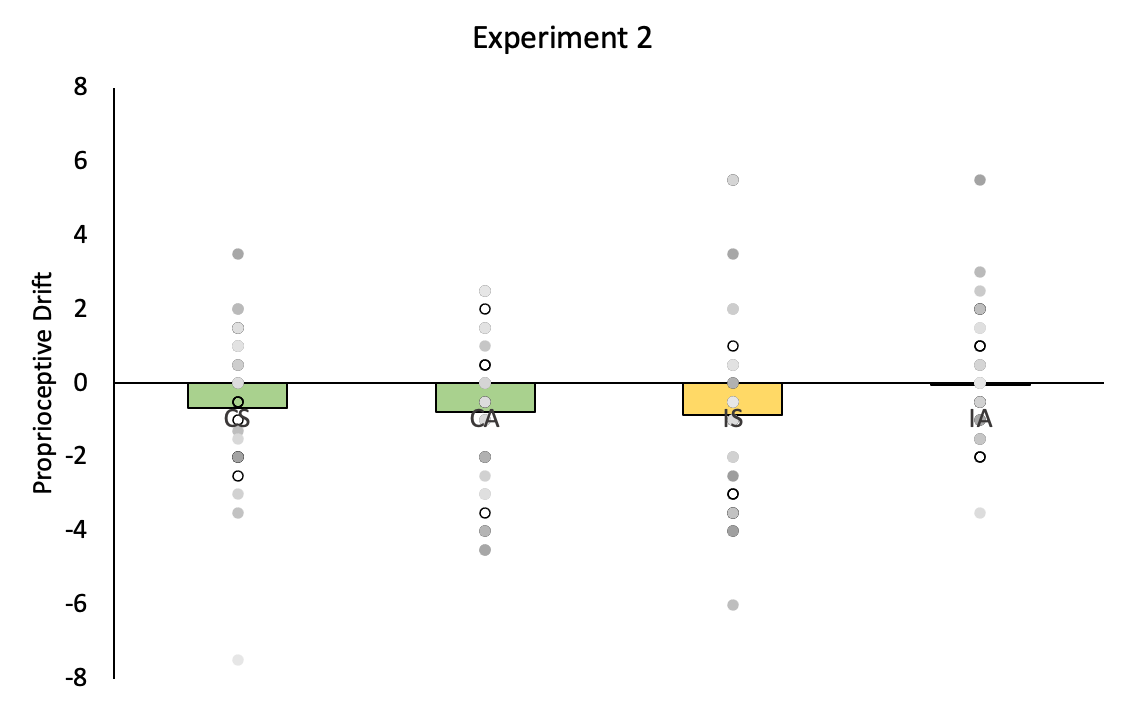


**Supplementary Figure 2.** Mean scores and individual data point distribution for the proprioceptive drift for Experiment 1 (CW = congruent warm; IW = incongruent warm; CC = congruent cold; IC = incongruent cold) and 2 (CS = congruent synchronous; CA = congruent asynchronous; IS = incongruent synchronous; IA = incongruent asynchronous). See also Figure 1b in the main text.


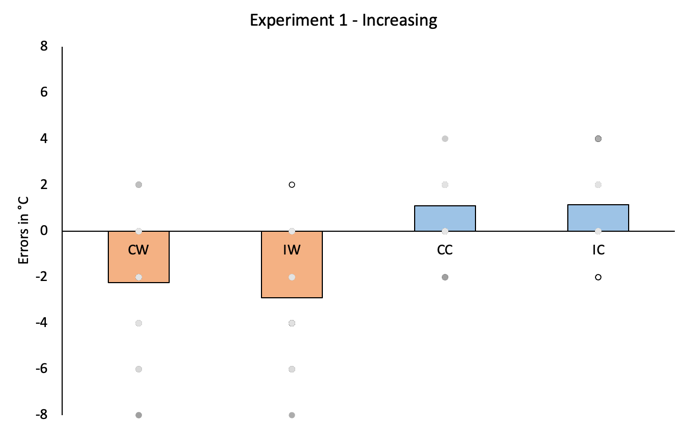


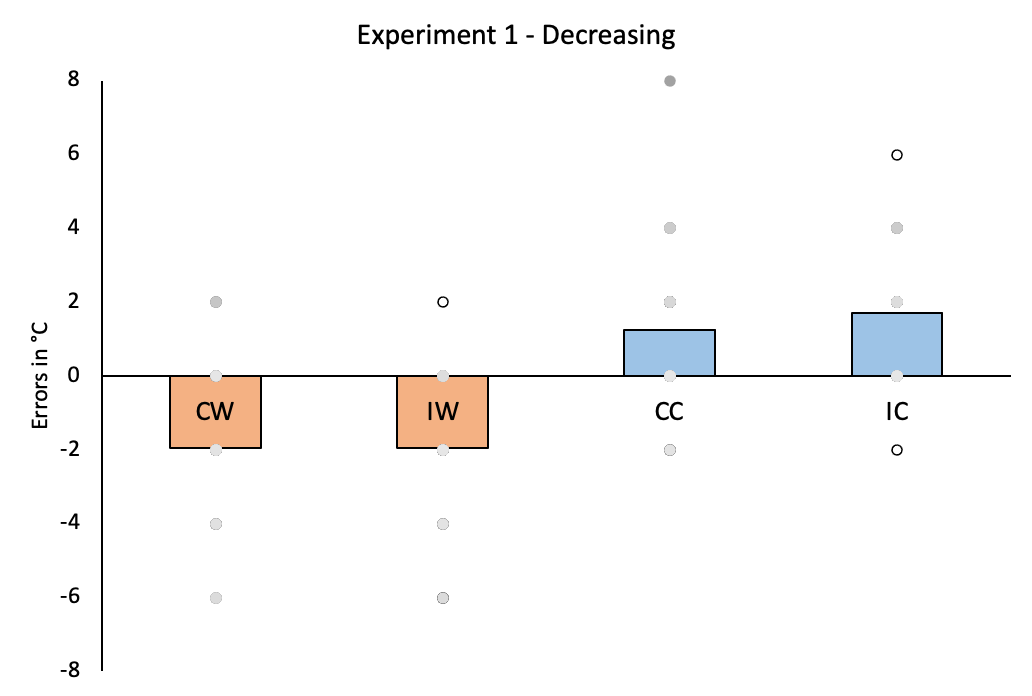


**Supplementary Figure 3**. Mean scores and individual data point distribution for performance in the thermal matching task for Experiments 1 (CW = congruent warm; IW = incongruent warm; CC = congruent cold; IC = incongruent cold). See also Figure 2 in the main text.


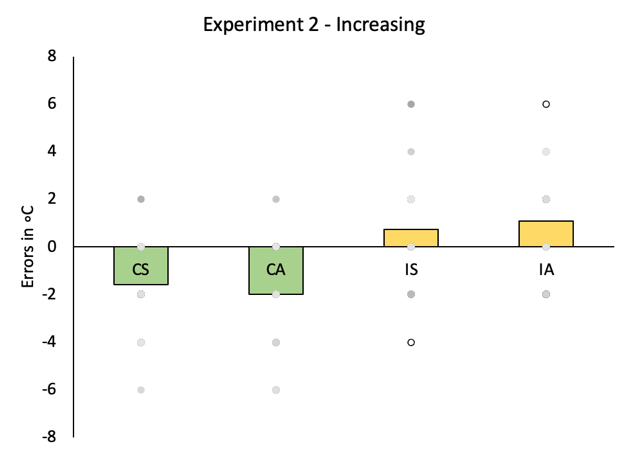

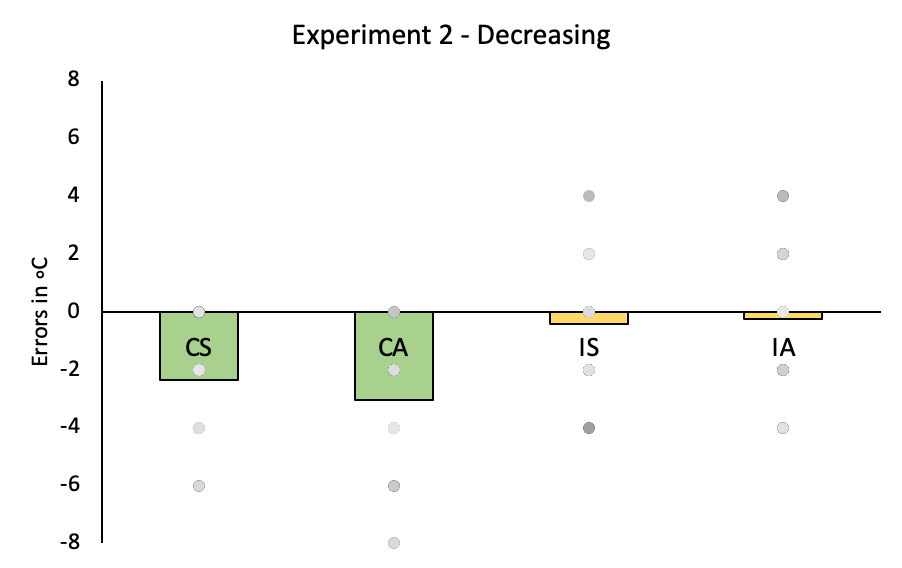


**Supplementary Figure 4**. Mean scores and individual data point distribution for performance in the thermal matching task for Experiments 2 (CS = congruent synchronous; CA = congruent asynchronous; IS = incongruent synchronous; IA = incongruent asynchronous). See also Figure 3 in the main text.
