# Supplementary material for: Visuo-thermal congruency modulates the sense of body ownership
Source: Commun Biol. 2022 Jul 22;5:731. doi: 10.1038/s42003-022-03673-6 (PMC9307774; doi:10.1038/s42003-022-03673-6)
Supplement: Supplementary file 3 — Description of Additional Supplementary Files [file 42003_2022_3673_MOESM3_ESM.pdf]

File name: Supplementary Data 1

Description: The processed data that support the findings of this study are available as Supplementary Data 1.
