# Supplementary material for: Visuo-thermal congruency modulates the sense of body ownership
Source: Commun Biol. 2022 Jul 22;5:731. doi: 10.1038/s42003-022-03673-6 (PMC9307774; doi:10.1038/s42003-022-03673-6)
Supplement: Supplementary file 5 — Reporting summary [file 42003_2022_3673_MOESM5_ESM.pdf]

nature portfolio

Corresponding author(s): Laura Crucianelli

Last updated by author(s): May 16, 2022

Reporting Summary

Nature Portfolio wishes to improve the reproducibility of the work that we publish. This form provides structure for consistency and transparency in reporting. For further information on Nature Portfolio policies, see our Editorial Policies and the Editorial Policy Checklist.

Statistics

For all statistical analyses, confirm that the following items are present in the figure legend, table legend, main text, or Methods section.

n/a	Confirmed	
☐	☒	The exact sample size (*n*) for each experimental group/condition, given as a discrete number and unit of measurement
☐	☒	A statement on whether measurements were taken from distinct samples or whether the same sample was measured repeatedly
☐	☒	The statistical test(s) used AND whether they are one- or two-sided<br>*Only common tests should be described solely by name; describe more complex techniques in the Methods section.*
☐	☒	A description of all covariates tested
☐	☒	A description of any assumptions or corrections, such as tests of normality and adjustment for multiple comparisons
☐	☒	A full description of the statistical parameters including central tendency (e.g. means) or other basic estimates (e.g. regression coefficient) AND variation (e.g. standard deviation) or associated estimates of uncertainty (e.g. confidence intervals)
☐	☒	For null hypothesis testing, the test statistic (e.g. F, t, r) with confidence intervals, effect sizes, degrees of freedom and P value noted<br>*Give P values as exact values whenever suitable.*
☐	☒	For Bayesian analysis, information on the choice of priors and Markov chain Monte Carlo settings
☐	☒	For hierarchical and complex designs, identification of the appropriate level for tests and full reporting of outcomes
☐	☒	Estimates of effect sizes (e.g. Cohen's *d*, Pearson's *r*), indicating how they were calculated

Our web collection on statistics for biologists contains articles on many of the points above.

Software and code

Policy information about availability of computer code

Data collection	Data were collected using the custom-made software of the Somedic MSA Thermal Stimulator (Somedic SenseLab AB, Sweden). Temperature of the skin was recorded using thermal sensors (Biopac MP150, Goleta, CA,United States) and visualised with the software AcqKnowledge (version 5.0).
Data analysis	Data were analysed using IBM SPSS Statistics for Windows, Version 26.0. Armonk, NY: IBM Corp.

For manuscripts utilizing custom algorithms or software that are central to the research but not yet described in published literature, software must be made available to editors and reviewers. We strongly encourage code deposition in a community repository (e.g. GitHub). See the Nature Portfolio guidelines for submitting code & software for further information.

Data

Policy information about availability of data

All manuscripts must include a data availability statement. This statement should provide the following information, where applicable:

- Accession codes, unique identifiers, or web links for publicly available datasets
- A description of any restrictions on data availability
- For clinical datasets or third party data, please ensure that the statement adheres to our policy

The processed data that support the findings of this study are available as Supplementary Data.

Field-specific reporting

Please select the one below that is the best fit for your research. If you are not sure, read the appropriate sections before making your selection.

☐ Life sciences ☒ Behavioural & social sciences ☐ Ecological, evolutionary & environmental sciences

For a reference copy of the document with all sections, see nature.com/documents/nr-reporting-summary-flat.pdf

Behavioural & social sciences study design

All studies must disclose on these points even when the disclosure is negative.

Study description	Data collected from this study were behavioural, as quantified by means of rating scales and behavioural task performance. We also used questionnaires data.
Research sample	In Experiment 1, forty participants (24 women, Mean age = 26.67; SD = 4.72) and in Experiment 2, thirty-three participants (16 women; Mean age = 26.39; SD = 5.03) were recruited using social media and advertising in the Karolinska Institutet campus. Inclusion criteria were being 18-40 years old and being right-handed. Exclusion criteria were having history of any psychiatric or neurological conditions, taking any medications, having sensory or health conditions that might result in skin conditions (e.g., psoriasis), having any scars or tattoos on their left forearm or hand.
Sampling strategy	A priori power analysis (G*Power 3.1, Faul et al., 2007) based on previous studies using within-subjects design in the RHI (e.g., Crucianelli et al., 2018; Radziun & Ehrsson, 2018) suggested that the present sample provides enough power to detect our effects of interest (power 0.92; $\alpha = 0.05$, effect size d = 0.6, two-tailed).
Data collection	Data were collected using behavioural methods, which were measured using pen and paper and a laptop for the questionnaires. Physiological measurements were collected with the Biopac and the thermal stimulator (see above). No one was present in the experimental room besides the participant and the researcher. The researcher was not blind to the experimental conditions and the study hypothesis.
Timing	Data collection for Experiment 1 was conducted between November 2019 and January 2020. Data collection for Experiment 2 was conducted between June 2020 and July 2020.
Data exclusions	No data were excluded from the analyses.
Non-participation	No participants dropped out/declined participation.
Randomization	Participants were no allocated to experimental groups. The order of experimental conditions in the rubber hand illusion was fully randomized.

Reporting for specific materials, systems and methods

We require information from authors about some types of materials, experimental systems and methods used in many studies. Here, indicate whether each material, system or method listed is relevant to your study. If you are not sure if a list item applies to your research, read the appropriate section before selecting a response.

Materials & experimental systems

n/a	Involved in the study	
☒	☐	Antibodies
☒	☐	Eukaryotic cell lines
☒	☐	Palaeontology and archaeology
☒	☐	Animals and other organisms
☐	☒	Human research participants
☒	☐	Clinical data
☒	☐	Dual use research of concern

Methods

n/a	Involved in the study	
☒	☐	ChIP-seq
☒	☐	Flow cytometry
☒	☐	MRI-based neuroimaging

Human research participants

Policy information about studies involving human research participants

Population characteristics	Our sample consisted of forty participants (24 women, Mean age = 26.67; SD = 4.72) for Experiment 1 and thirty-three participants (16 women; Mean age = 26.39; SD = 5.03) for Experiment 2. Inclusion criteria were being 18-40 years old and being right-handed. Exclusion criteria were having history of any psychiatric or neurological conditions, taking any medications, having sensory or health conditions that might result in skin conditions (e.g., psoriasis), having any scars or tattoos on their left forearm or hand. Upon arrival and after signing the consent form, participants provided demographic

	information and completed the Body Perception Questionnaire (BPQ short version, Porges, 1993) as measures of interoceptive sensibility (Mean BPQ = 29.36; SD = 10.62).
Recruitment	Participants were recruited using social media and advertising in the Karolinska Institutet campus.
Ethics oversight	The study was approved by the Swedish Ethical Review Authority. The study was conducted in accordance with the provisions of the Declaration of Helsinki 1975, as revised in 2008.

Note that full information on the approval of the study protocol must also be provided in the manuscript.
