# Supplementary material for: Visuo-thermal congruency modulates the sense of body ownership
Source: Commun Biol. 2022 Jul 22;5:731. doi: 10.1038/s42003-022-03673-6 (PMC9307774; doi:10.1038/s42003-022-03673-6)
Supplement: Supplementary file 6 — Model release form [file 42003_2022_3673_MOESM6_ESM.pdf]

Model Release Form

SPRINGER NATURE

Details of intended publication (if known):

Journal Name:	Communication Biology
Title of the Article:	Visuo-thermal congruency modulates the sense of body ownership

Model Details:

Name ("the Model"):	Dominika Radziun
Address:	Department of Neuroscience, Solnavägen 9, 171 65 Stockholm, Sweden
Telephone Number	(+46) 8 524 87 989
Photographs taken at (location):	Department of Neuroscience, Solnavägen 9, 171 65 Stockholm, Sweden
Description of photograph/s (or attached copies if available):	The photographs show the experimental set-up

To: Springer Nature Limited (the "Publisher")

In exchange for valuable consideration, I, the Model, hereby grant to you, the Publisher, permission to photograph me (if applicable). I further irrevocably give you, the Photographer, and your licensees and assignees, the right to publish, reproduce or otherwise cause to be transmitted the photograph(s) of me in any publication (commercial or otherwise), portfolio, public display or similar, in any medium throughout the world and I hereby waive any right to privacy in relation to the images.

I hereby waive any right I may otherwise have to inspect or approve a finished product or copy embodying my image. I understand that the images may be cropped and that there is no guarantee of my inclusion in the images.

I am at least 18 years of age and am competent to contract in my own name. I have read this release before signing below and I fully understand the contents, meaning, and impact of this release. This Agreement shall be governed by and construed in accordance with the laws of England and Wales.

Signature [Handwritten signatures only]:

Dominika Radziun

Print Name:	Dominika Radziun
Date:	19/05/2022

If the person signing is under age 18, there must be consent by a parent or guardian, as follows:

I hereby certify that I am the parent or guardian of Click here to enter text. , named above, and do hereby give my consent without reservation to the foregoing on behalf of this person.

Signature (Parent/Guardian) [Handwritten signatures only]:	
Print Name (Parent/Guardian):	Click here to enter text.
Date:	Click here to enter text.

Office use only:

Springer Nature Limited.
Registered office: The Campus, 4 Crinan Street, London, N1 9XW, UK. Company number 785998.
